# Interaction of coronavirus E protein with BRD2 plays important regulatory roles in viral replication and induction of pro-inflammatory response

**DOI:** 10.1128/jvi.02201-25

**Published:** 2026-03-03

**Authors:** Shumin Li, Ding Xiang Liu

**Affiliations:** 1 Zhaoqing Branch Center of Guangdong Laboratory for Lingnan Modern Agricultural Science and Technology, Zhaoqing, Guangdong, China; 2 Integrative Microbiology Research Centre, South China Agricultural University, Guangzhou, Guangdong, China; 3 Agro-biological Gene Research Center of Guangdong Academy of Agricultural Sciences, State Key Laboratory of Swine and Poultry Breeding Industry, Guangzhou, China; Loyola University Chicago - Health Sciences Campus, Maywood, Illinois, USA

**Keywords:** coronavirus, E protein, BRD2, pro-inflammatory cytokines and chemokines

## Abstract

The devastating COVID-19 pandemic has urged continuous efforts to uncover innovative intervention measures through a thorough understanding of viral and associated host protein functions. In this study, we investigate the interaction of coronavirus E protein with BRD2, a member of the bromodomain and extra-terminal (BET) protein family, and the mechanism underlying the regulatory role of BRD2 in promoting pro-inflammatory responses during coronavirus infection, based on the reported potential interaction of BRD2 with severe acute respiratory syndrome coronavirus-2 (SARS-CoV-2) E protein and our transcriptomic assay. We show that coronavirus infection significantly upregulates the expression of pro-inflammatory cytokines and BET family genes and activates the NF-κB pathway, although the ion channel (IC) activity of the avian infectious bronchitis virus (IBV) E protein only minimally affects the upregulation of BRD2 transcription. Furthermore, BRD2 was verified to interact with the E protein from IBV, SARS-CoV, and SARS-CoV-2, via the BDII-containing C-terminal region of BRD2 and the C-terminal region of the E protein. This interaction resulted in the partial translocation of BRD2 from the nucleus to the cytoplasm in IBV-infected cells and its close association with viral particles. Overexpression of BRD2 and IBV E protein independently induced the expression of pro-inflammatory cytokines, and a synergistic role was demonstrated in cells co-expressing the two proteins. BRD2 was further found to activate the NF-κB signaling pathway and enhance the expression of pro-inflammatory cytokines IL-6, IL-8, and TNF-α at the mRNA level. Conversely, knockdown of BRD2 promotes viral replication and apoptosis. These findings underscore the interaction between BRD2 and E protein, elucidating their roles in regulating coronavirus replication and promoting the pro-inflammatory response.

**IMPORTANCE:**

Severe coronavirus infections usually result in excessive secretion of inflammatory cytokines and chemokines, leading to cell death and ultimately collapse of the immune system with fatal outcomes. So far, intensive studies on host-pathogen interactions have partially elucidated the immunopathogenesis of COVID-19 and other coronavirus infections. This study reveals that coronavirus infection induces the expression of BRD2 and activates the NF-κB pathway. BRD2 interacts with coronavirus E protein, and the two proteins work together to play a synergistic role to promote pro-inflammatory response during coronavirus infection. Furthermore, BRD2 was shown to independently activate the NF-κB pathway and enhance the expression of IL-6, IL-8, TNF-α, and several other proteins related to the pro-inflammatory and inflammatory responses. The interaction of BRD2 with coronavirus E protein and its regulatory role in inflammation would make it a potential therapeutic target for severe coronavirus infections.

## INTRODUCTION

Coronaviruses (CoVs) are enveloped, positive-stranded RNA viruses that infect both animals and humans, representing an enormous public health and economic challenge globally (1). Several human CoVs, including severe acute respiratory syndrome coronavirus (SARS-CoV), Middle East respiratory syndrome coronavirus (MERS-CoV), and SARS-CoV-2, cause severe respiratory diseases and fatalities (2–4). Animal CoVs, such as avian infectious bronchitis virus (IBV) and porcine epidemic diarrhea virus (PEDV), pose immense economic threats to poultry and pig industries (5, 6). CoVs have a large RNA genome of 26–32 kb in length and feature four key structural proteins, including the spike (S) glycoprotein crucial for viral entry and immunity (7), the membrane (M) glycoprotein functioning in viral budding (8), the nucleocapsid (N) protein mediates viral assembly and budding by compacting the genomic RNA into a beads-on-a-string chain (9), and the envelope (E) protein essential for virus assembly (10).

E protein is a small (8–12 kDa), hydrophobic membrane protein found in low quantities in the virion (10). It plays a key role in virus-host interactions and inflammation during infection. Acting as a viroporin, the E protein oligomerizes into a pentameric structure, forming ion channels to facilitate virus-host interactions (11). This ion channel activity plays multiple roles in viral replication and regulation of host responses to CoV infection, including viral assembly, budding, envelope formation, and inflammasome activation (12). Examples include that SARS-CoV and SARS-CoV-2 E protein suppresses the IRE1 pathway, lowering cell death and raising inflammation (13), and the ion channel activity of IBV E protein enhanced the ER stress response and pro-inflammatory cytokine production (14). Some CoV E proteins also encode PDZ-binding motifs (PBMs) at their C-terminal end to interact and regulate host response to viral infection, such as to activate the p38 MAPK and inflammatory cytokines overexpression (15).

The bromodomain and extra-terminal (BET) proteins, including BRD2, BRD3, BRD4, and BRDT (16), are a unique family of bromodomain proteins that act as transcriptional mediators and function as chromatin readers by binding to acetylated lysine residues on histones and non-histone proteins through their conserved bromodomains (16). In addition, proteins in this family modulate gene expression epigenetically via histone modification, playing a key role in regulating inflammatory responses (17, 18). Among the BET proteins, BRD2 was initially identified as a regulator of cell cycle and transcription (19, 20). Its regulatory role in inflammation by binding to acetylated histones at cytokine gene promoters was revealed by a couple of subsequent studies. For example, BRD2 depletion in macrophages reduced the pro-inflammatory cytokine production (21), and elevated BRD2 in lung vascular cells in pulmonary arterial hypertension (PAH) patients increased IL-6, IL-8, and TNF-α levels (22). BRD2 may also activate inflammation by directly binding acetylated NF-κB/RelA, not just through histone interactions (21).

The BET family of proteins has been shown to play certain regulatory roles in the replication and pathogenicity of SARS-CoV-2. SARS-CoV-2 E protein was found to bind to BRD2 and BRD4 to regulate the host immune response, suggesting BET proteins as targets for host-directed therapies (23). BRD2 was also found to increase the expression of SARS-CoV-2 receptor angiotensin-converting enzyme 2 (ACE2) and genes involved in inflammation and interferon response during infection (24). On the contrary, E protein may compete with BET proteins via these interactions (BRD2 and BRD4) for the same binding sites on histones, potentially disrupting host transcription (25). So far, the direct interaction of E protein with BRD2 and the potential role of this interaction in CoV replication and pathogenesis are yet to be systematically investigated. This study explores the role of BRD2 in CoV infection, focusing on its regulation of inflammation and interaction with viral E proteins. We found that BET proteins are activated in most cell types infected with IBV, PEDV, and two low-pathogenic human CoVs, HCoV-229E and HCoV-OC43, and modulate pro-inflammatory cytokine transcription during infection. The interaction of the E protein from different CoVs with BRD2 plays a synergistic role in regulating pro-inflammatory response, highlighting BRD2 as a promising common therapeutic target for CoVs.

## MATERIALS AND METHODS

### Cell culture and virus

Vero, 293T, IPEC-J2, DF1, and HeLa cells were cultured in DMEM (10% FBS, 1% penicillin-streptomycin), H1299 cells were maintained in RPMI1640 (8% FBS) at 37°C with 5% CO₂.

IBV-p65 strain (GenBank: DQ001339) was generated by adapting the Beaudette strain (ATCC VR-22) to Vero cells (26). HCoV-229E (accession No. KU291448.1) (27), HCoV-OC43 (accession No. KU131570.1) (28), and PEDV virulent strain DR13 (accession No. JQ023162) (29) were obtained from ATCC.

For virus propagation, Vero cells (IBV-p65, PEDV) or MRC-5 cells (HCoVs) were infected with respective viruses at a multiplicity of infection (MOI) of ~0.1 in plain DMEM until cytopathic effects appeared. Lysates were freeze-thawed three times, clarified (3,000 × *g*, 10 min, 4°C), and stored at −80°C. Viral titers were determined by plaque/TCID50 assays. In all experiments, cells were infected with the specified virus at an MOI of ~2, with mock controls receiving uninfected lysates.

### Antibodies, chemicals, and reagents

Antibodies against BRD2 (#5848), NF-κB p65 (#8242), Phospho-NF-κB p65 (#3033), IκBα (#4814), Phospho-IκBα (#5209), IL-6 (#12912), FLAG-Tag (#8146), GFP-Tag (#2956), myc-Tag (#2276), H3 (#4499), α-Tubulin (#3873), and β-actin (#4967) were from Cell Signaling Technology. Antibodies against HCoV-OC43 N (40643-T62) and HCoV-229E N (40640-T62) were from SinoBiological. Secondary antibodies (Alexa Fluor 488, ab150077; Alexa Fluor 647, ab150115) were from Abcam. Anti-IBV N antisera were generated in rabbits as previously described (30). Poly (I:C) and lipopolysaccharide (LPS) (Sigma) were dissolved in RNase-free water (2 mg/mL) and stored at −80°C.

### Transcriptomic analysis

Transcriptomic analysis was conducted by Biomarker Technologies Co., Ltd. in Beijing, China, essentially as described in a previously published report (31).

### Plasmid construction and transfection

Human BRD2 cDNA (NM_001113182.3) was amplified from H1299 cells using primer pair 5′GACTCACTATAGGGCGAATTCCCACCATGCTGCAAAACGTGACTC3′ and 5′AAGATCTGGTACCGAGCTCCTTAGCCTGAGTCTGAATCACTG3′, and cloned into pXJ40-FLAG (*Bam*HI*/Pst*I) and pXJ40-myc (*Bam*HI/*Kpn*I) via homologous recombination. IBV E cDNA was amplified from infected H1299 cells using primer pair 5′CCGGAATTCCCACCATGAATTTATTGAATAAGTCG3′ and 5′CGGGGATCCAGAGTACAATTTGTCTCGTTGG3′, SARS-CoV E cDNA was amplified using primer pair 5′CGACTCACTATAGGGCGAATTCCCACCATGTACTCATTCGTTTC3′ and 5′AAGATCTGGTACCGAGCTCCTCAGTGGGCCAGAGCCGCA3′. SARS-CoV-2 E cDNA was amplified using primer pair 5′ACGACTCACTATAGGGCGAATTCCCACCATGTACTCATTCGTTTCG3′ and 5′CGTCGTCCTTGTAGTCGGATCCGACCAGAAGATCAGGA3′. The amplified products were cloned into pXJ40-FLAG (*Bam*HI/*Eco*RI) and pXJ40-GFP (*Pst*I*/Eco*RI), respectively.

IBV E-T16A mutant was generated using primer pair 5′GTAATACGACTCACTATAGGGCGAATTCCCACCATGAATTTATTGAATAAGTCGCTAGAGGAG3′ and 5′GAAGCTCGAGTCAAGAGTACAATTTGTC3′, and IBV E-A26F mutant was generated using primers 5′GTAATACGACTCACTATAGGGCGAATTCCCACCATGAATTTATTGAATAAGTCGCTAGAGGAG3′ and 5′ATCGTCGTCCTTGTAGTCGGATCCAGAGTACAATTTGTCTCGTTGG3′. Both mutations disrupt IBV E ion channel activity (32).

H1299 and 293T cells at 60%–70% confluency were transfected with 2 μg plasmid DNA using TransIntro EL (Transgen Biotech) in Opti-MEM and incubated for 6–8 h. The transfection medium was replaced with complete 5% FBS medium. At 24 h post-transfection, cells were infected with IBV (MOI ~2) or mock-treated, and samples were collected at designated time points post-infection.

### Generation of stable shRNA-transfected cell lines and cell proliferation assay

The lentivirus-based BRD2 short hairpin RNA (shBRD2) sequence and a negative control short hairpin RNA (shNC) sequence were designed and cloned into the pLKO.1-puro vector plasmid by Tsingke biotechnology company (Beijing, China). Lentiviruses were produced by co-transfecting HEK 293T cells with the shRNA vectors and helper plasmids (pSPAX2/pMD2G, 4:3:1 ratio). Supernatants containing viral particles were collected after 48–72 h and used to infect H1299 and Vero cells for 8 h post-infection, the medium was replaced, and puromycin selection (2 μg/mL for H1299, 5 μg/mL for Vero) was applied after 2 days. Stable shRNA-expressing cells were then obtained for experiments.

H1299-shBRD2/shNC and Vero-shBRD2/shNC (5 × 10^3^) cell lines were seeded on 96-well plates and incubated at 37°C for 16 h. Then, 10 µL of CCK solution was added to the plates and incubated at 37°C for 2 h. The absorbance at 450 nm was detected by an automatic microplate reader.

### RNA interference

BRD2 siRNA (+): 5′GCCCUCUUUACGUGAUUCAAATT3′, EGFP siRNA (+): 5′GCUGACCCUGAAGUUCAUCTT3′ were purchased from Sangon Biotech. For transfection, H1299 cells were seeded in 12-well plates and grown to 40%–50% confluence. Each well received a mix of 5 µL siRNA (20 µM) and 2.5 µL TransIntro EL (Transgen Biotech). After 36–48 h, cells were infected with IBV at an MOI of ~2 or mock-infected. Samples were collected at specified time points. Three sets of siRNA duplexes were used in all knockdown experiments, and the representative data from the siRNA duplex with the best knockdown efficiency are presented.

### RNA extraction and RT-qPCR analysis

Total RNA was extracted using TRIzol reagent (Invitrogen) following standard chloroform-isopropanol precipitation. RNA pellets were washed with 70% ethanol and resuspended in RNase-free water. Reverse transcription was performed using FastKing gDNA Dispelling RT SuperMix (Tiangen) with 2 μg RNA in 20 μL reactions (42°C for 15 min, 95°C for 3 min). cDNA was diluted 20× for subsequent analysis. qPCRs (20 μL) contained SYBR Green PreMix (Tiangen), diluted cDNA, 0.6 μL each of 10 μM primers, and ROX reference dye. Cycling conditions: 50°C (3 min), 95°C (3 min), then 40 cycles of 95°C (5 s) and 60°C (30 s). Relative expression was calculated using the ΔΔCT method with GAPDH normalization.

The following qPCR primer pairs were used. GAPDH, 5′CTGGGCTACACTGAGCACC3′ and 5′AAGTGGTCGTTGAGGGCAATG3′; IBVgRNA, 5′GTTCTCGCATAAGGTCGGCTA3′ and 5′GCTCACTAAACACCACCAGAAC3′; PEDV gRNA, 5′CTGAGCAAATTCGCTGGCG3′ and 5′AACACCCTCAGTACGAGTCC3′; 229E gRNA, 5′TAGGTTTTGACAAGCCTCAGGAAAAAGA3′ and 5′ACGAGCAAGACTCTTGGCAG3′; OC43 gRNA, 5′CTATCTGGGAACAGGACCGC3′ and 5′TTGGGTCCCGATCGACAATG3′; BRD2, 5′GAGGTGTCCAATCCCAAAAAGC3′ and 5′ATGCGAACTGATGTTTCCACA3′; BRD3, 5′TGCAAGCGAATGTATGCAGGA3′ and 5′CATCTGGGCCACTTTTTGTAGAA3′; BRD4, 5′ACCTCCAACCCTAACAAGCC3′ and 5′TTTCCATAGTGTCTTGAGCACC3′; BRDT, 5′CTGTTGACGTTAATGCTTTGGG3′ and 5′ CACAACTTCGTGATCTGGAGG3′; TNF-α, 5′GAGGCCAAGCCCTGGTATG3′ and 5′CGGGCCGATTGATCTCAGC3′; IL-6, 5′GTGCAAATGAGTACAAAAGTCCTGA3′ and 5′GTTCTGCGCCTGCAGCTTC3′; IL-8, 5′AAGACGTACTCCAAACCTATCCAC3′ and 5′TCTGTATTGACGCAGTGTGGTC3′.

### SDS-PAGE and western blot analysis

Cells were harvested by centrifugation (16,000 × *g* for 1 min) and lysed in RIPA buffer. Clarified lysates or culture supernatants were mixed with 5× Laemmli buffer (33). After heating at 95°C for 5 min and centrifugation, equal protein amounts were separated by SDS-PAGE (Bio-Rad Mini-PROTEAN) and transferred to nitrocellulose membranes (0.2 μm, Bio-Rad Trans-Blot). Membranes were blocked with 5% skim milk/TBST at room temperature for 1 h, then incubated overnight at 4°C with primary antibodies (1 μg/mL in TBST + 3% BSA). After TBST washes, membranes were probed with goat anti-rabbit/mouse IgG secondary antibodies (Licor, 1:10,000) at room temperature for 2 h. Signals were detected using an Azure c600 Imager and quantified with AzureSpot software. Experiments were repeated ≥3 times with consistent results. Image Presentation: In this manuscript, when results from parallel measurements of the same sample batch are presented in different figures, the loading control or key reference bands may be reused, as explicitly stated in the relevant figure legends. All image adjustments were applied globally.

### Co-Immunoprecipitation

Lyse transfected cells (six-well plate) with 300 µL RIPA buffer/well, add 3 µL protease inhibitor, mix, and rotate at 4°C for 15 min. After centrifugation at 12,000 rpm for 10 min, transfer the supernatant to a tube with washed beads. Adjust volume to 1 mL with lysis buffer, add 10 µL protease inhibitor, and incubate overnight at 4°C. After centrifugation at 8,200 *× g*, 4°C for 30 s, discard supernatant, and wash pellets 3× with 1 mL lysis buffer. Afterward, total cell lysates and precipitates were boiled with sample buffer and then analyzed by Western blot.

### Immunofluorescence and confocal microscopy

The Vero cells cultured in 48-well plates to confluency were infected with IBV-p65 at an MOI of ~2. At 20 h post-infection, the cells were fixed with ice-cold 100% methanol (15 min, −20°C), rinsed three times with 1 *×* PBS for 5 min each. The cells were then incubated with blocking buffer at room temperature for 1 h, diluted primary antibody was added and incubated at 4°C overnight, and rinsed three times with 1 *×* PBS for 5 min each. After incubation with diluted secondary antibody for 1–2 h at room temperature in the dark, the cell nuclei were stained by adding 10 µg/mL Hoechst 33342 and incubated at room temperature for 5–10 min, rinsed three times with 1 *×* PBS for 5 min each. Finally, images were acquired with a fluorescence microscope (or a confocal microscope).

### Nuclear and cytoplasmic protein extraction

Confluent Vero cells infected with IBV were washed with PBS at different time points post-infection, and cell pellets were collected. Nuclear and cytoplasmic proteins were fractionated using the nuclear and cytoplasmic protein extraction kit (Beyotime), added to 5× SDS, heated at 95°C for 3–5 min, and analyzed by SDS-PAGE and Western blot.

### Purification of IBV

Confluent monolayers of Vero cells were infected with IBV strain Beaudette at an MOI of ~2. After incubation at 37°C until 100% CPE was observed, the virus was harvested from supernatants and purified by ultracentrifugation. Briefly, 200 mL of supernatant from 40 infected cell dishes (100 mm diameter) was layered onto six tubes, each containing 5 mL of 20% (wt/vol) sucrose in PBS, and centrifuged at 35,000 × *g* for 3 h at 4°C. The pelleted virus was resuspended in 1 mL PBS, layered onto a 13 mL 20%–60% linear sucrose gradient, and centrifuged again at 35,000 × *g* for 4 h at 4°C. Finally, 13 fractions were collected from the gradient and analyzed by SDS-PAGE.

### Statistical analysis

The two-way ANOVA method was used to analyze the significant difference between the indicated sample and the respective control sample. Significance levels were presented by the *P*-value (ns, non-significant; *, *P* < 0.05; **, *P* < 0.01; ***, *P* < 0.001).

## RESULTS

### BET family genes are differentially induced by CoV infection

Our previous transcriptomics analysis has shown that IBV infection significantly upregulates host chromatin remodeling and inflammatory pathways (31). As the coronavirus E protein is known to play various immunomodulatory functions and was shown to interact with BET family proteins in a previously published proteomics study (23), we first set out to investigate the effects of coronavirus infection on the expression of BET family proteins and the potential interaction of E protein with these proteins. As summarized in Table 1, the transcription levels of most BET family genes were not significantly affected by IBV infection in H1299 cells infected with IBV at an MOI of ~2 or mock-treated for 16 h, based on the general mRNA expression profile of host genes differentially regulated by IBV infection obtained by transcriptomic analysis (31). However, compared with the mock control, the mRNA levels of BRD2 and BRD4 were induced by 2.4- and 1.5-fold, respectively (Table 1).

**TABLE 1:** Transcriptomic analysis of BET proteins in H1299 cells infected with IBV^*a*^

	Mock (FPKM)			IBV infection (FPKM)			Fold change	FDR
	1	2	3	1	2	3		
BRD2	0.921	0.947	1.067	2.063	2.495	2.565	2.390	<0.001
BRD3	13.109	13.491	12.663	13.088	6.986	7.722	0.538	0.002
BRD4	14.900	14.182	16.595	21.882	22.273	22.681	1.441	<0.001
BRDT	0.113	0.000	0.000	0.019	0.032	0.065	0.974	<0.001

^*a*^ FPKM, fragments per kilobase of transcript per million mapped reads; FDR, false discovery rate; BRD2, bromodomain 2; BRD3, bromodomain 3; BRD4, bromodomain 4; BRDT, bromodomain T.

**Fig 1:**
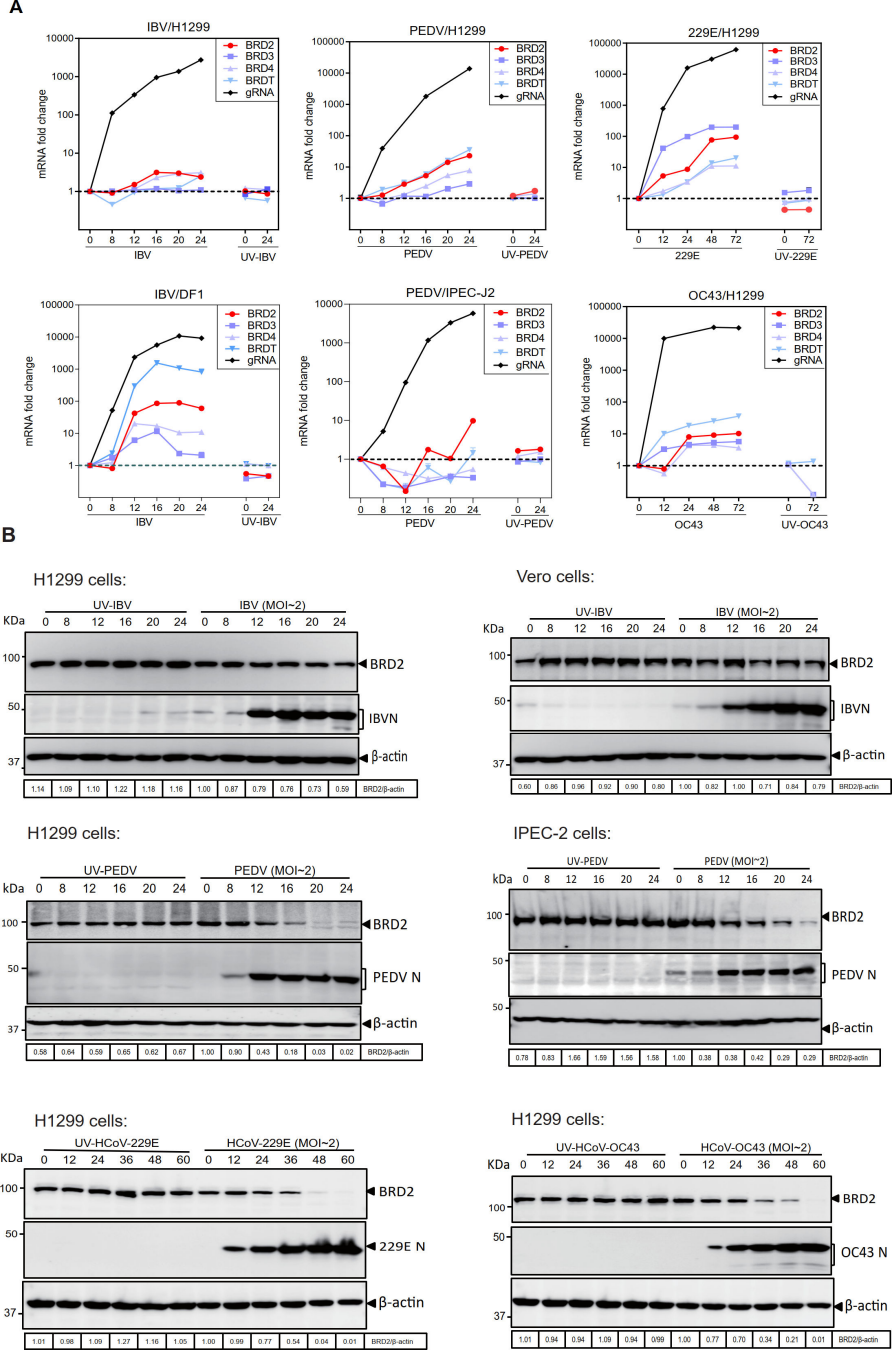
BET family genes are differentially induced by CoV infection. (**A**) H1299, DF1, IPEC-J2, and Vero cells were infected with IBV, PEDV, HCoV-OC43, or HCoV-229E (MOI ~2) or mock-treated with UV-inactivated viruses. Total RNA was extracted at the indicated time points, and RT-qPCR was performed to quantify viral genomic RNA (CoV gRNA) and BET family mRNAs (BRD2, BRD3, BRD4, and BRDT). Data were normalized to GAPDH mRNA (0 hpi) using the ΔΔCt method (ns, non-significant; *, *P*<0.05; **, *P* < 0.01; ***, *P* < 0.001). N.D., non-determined. (**B**) Cells were infected or mock-treated as in panel **A**, harvested at the indicated times, and analyzed by Western blot with the specified antibodies. β-actin served as a loading control. Protein ladder sizes (kDa) are shown.

To verify the transcriptomic analysis results, the temporal mRNA expression profiles of selected BET family genes were analyzed by time course infection experiments in cells infected with gammacoronavirus IBV, alphacoronavirus PEDV, and HCoV-229E, and betacoronavirus HCoV-OC43, respectively. The mRNA levels of BET family genes were consistent with the transcriptomic analysis results in H1299 cells infected with IBV and IPEC-J2 cells infected with PEDV (Fig. 1A). Nevertheless, the expression of BET family genes was significantly induced in H1299 cells infected with PEDV, HCoV-229E, and HCoV-OC43, and in DF1 cells infected with IBV, with more than 10 times upregulation (Fig. 1A).

Subsequently, the expression of BRD2 at the protein level was analyzed, showing slight decreased expression at late stages of IBV infection in H1299 and Vero cells (Fig. 1B). In H1299 and IPEC-J2 cells infected with PEDV and H1299 cells infected with HCoV-229E and HCoV-OC43, respectively, a significant downregulation of BRD2 expression at the protein level was observed in the late stages of infection (Fig. 1B). These data demonstrate that the differential induction of BRD2 at the mRNA level is not reflected at the protein expression, probably due to factors such as mRNA degradation, protein degradation, modification, folding, and other variables.

### Interaction of the C-terminal region of BRD2 with the C-terminal region of E protein

**Fig 2:**
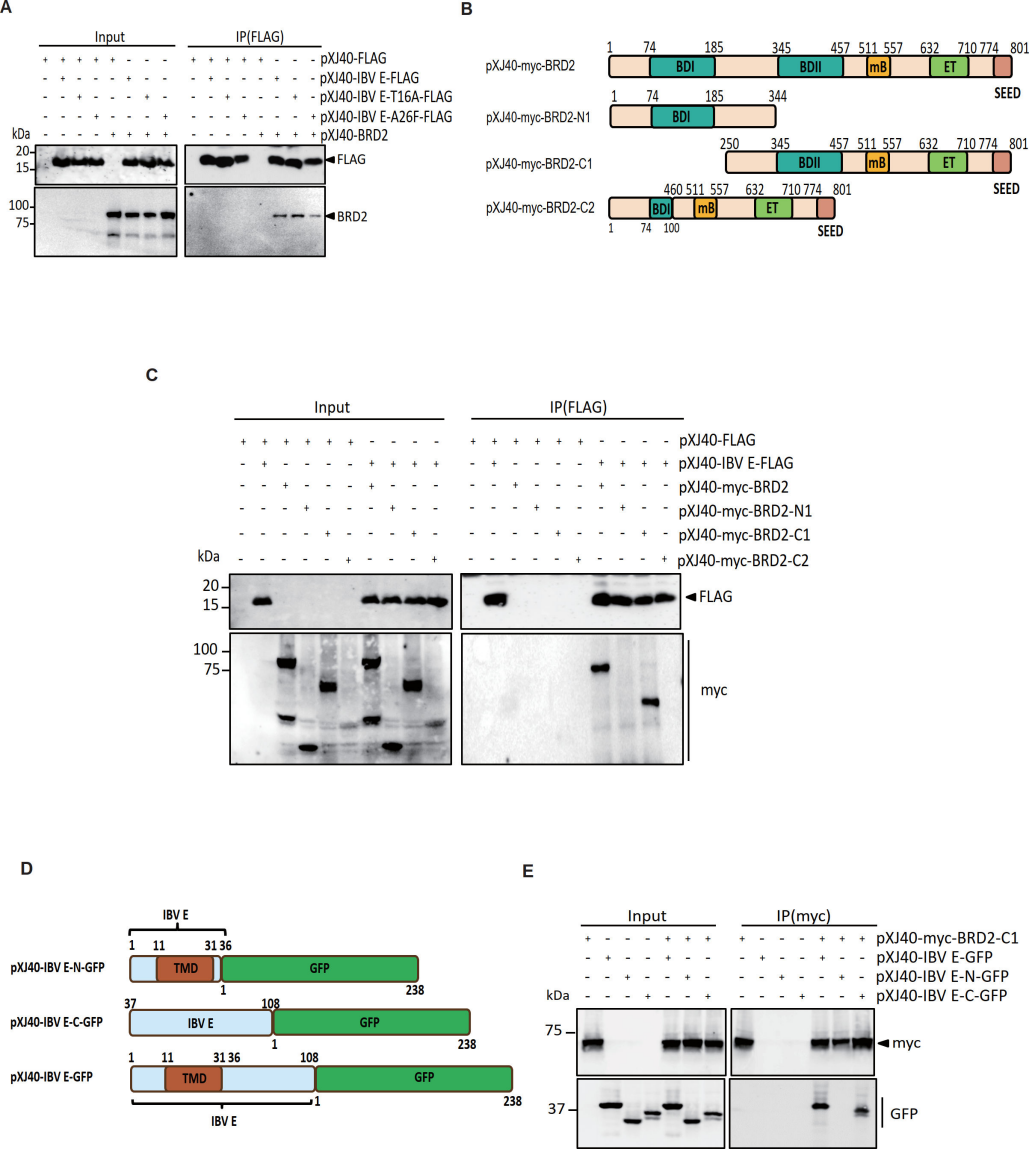
Interaction of the C-terminal region of BRD2 protein with the C-terminal region of CoV E protein. (**A**) 293T cells were transfected with pXJ40-FLAG, pXJ40-IBV E-FLAG, pXJ40-IBV E-T16A-FLAG, pXJ40-IBV E-A26F-FLAG, or pXJ40-BRD2. After 24 h, lysates were immunoprecipitated (IP) with anti-FLAG beads and analyzed by Western blot (anti-FLAG/anti-BRD2). Protein ladder sizes (kDa) are shown. Sizes of protein ladders in kDa were indicated on the left. (**B**) Diagram showing the putative functional domains in the full-length and three truncated BRD2 constructs, BRD2-N1, BRD2-C1, and BRD2-C2. (**C**) 293T cells were transfected with pXJ40-FLAG, pXJ40-IBV E-FLAG, or myc-tagged BRD2 constructs (full-length, N1, C1, C2). Lysates were immunoprecipitated with anti-FLAG and probed with anti-FLAG/anti-myc. Sizes of protein ladders in kDa were indicated on the left. (**D**) Diagram showing the putative functional domains in the full-length and two truncated IBV E constructs, IBV E-N, IBV E-C. (**E**) 2293T cells expressing myc-BRD2-C1 with GFP-tagged IBV E (full-length, N-, or C-terminus) were immunoprecipitated with anti-myc beads and blotted with anti-myc/anti-GFP. Sizes of protein ladders in kDa were indicated on the left.

In 2020, a large-scale proteomic analysis showed that SARS-CoV-2 E protein may interact with BRD2 and BRD4 proteins (23). Co-immunoprecipitation studies in HEK293T cells overexpressing tagged forms of E protein from IBV, SARS-CoV E, and SARS-CoV-2, respectively, and BRD2 proteins were performed to verify this interaction. IBV E protein and its two EIC mutants were all shown to interact with BRD2 (Fig. 2A). To define the specific interacting domain(s), three truncated BRD2 constructs were constructed based on the location of functional domains, including BRD2-N1 containing the BDI domain, BRD2-C1 containing the BDII domain, and BRD2-C2 containing a small portion of the BDI domain and the region downstream of the C-terminal part of the BDII domain (Fig. 2B). Co-immunoprecipitation experiment demonstrated that only BDII domain-containing BRD2-C1 can interact with IBV E (Fig. 2C). SARS-CoV E and SARS-CoV-2 E proteins were also shown interact with BRD2 (Fig. S1A). The specific site of interaction between these two CoV E proteins and BRD2 was also mapped to the BDII domain of BRD2, similar to IBV E protein (Fig. S1B and C).

To pinpoint the interacting region in the E protein, two truncated plasmids were constructed for the E protein from IBV, SARS-CoV, and SARS-CoV-2, respectively, based on the location of domains. Plasmid E-N contains the transmembrane domain (TMD) and plasmid E-C contains the C-terminal region without the TMD domain (Fig. 2D and Fig. S1D and E). Co-immunoprecipitation studies showed that the BDII domain predominantly interacts with the C-terminal region of the E proteins from IBV (Fig. 2E), SARS-CoV (Fig. S1F), and SARS-CoV-2 (Fig. S1G). These results confirm that the BRD2 protein interacts with the E protein from all three CoVs through the association of the BDII domain with the TMD-free C-terminal region of the E protein.

### Re-localization of BRD2 from the nucleus to the cytoplasm in CoV-infected cells and its association with virions

**Fig 3:**
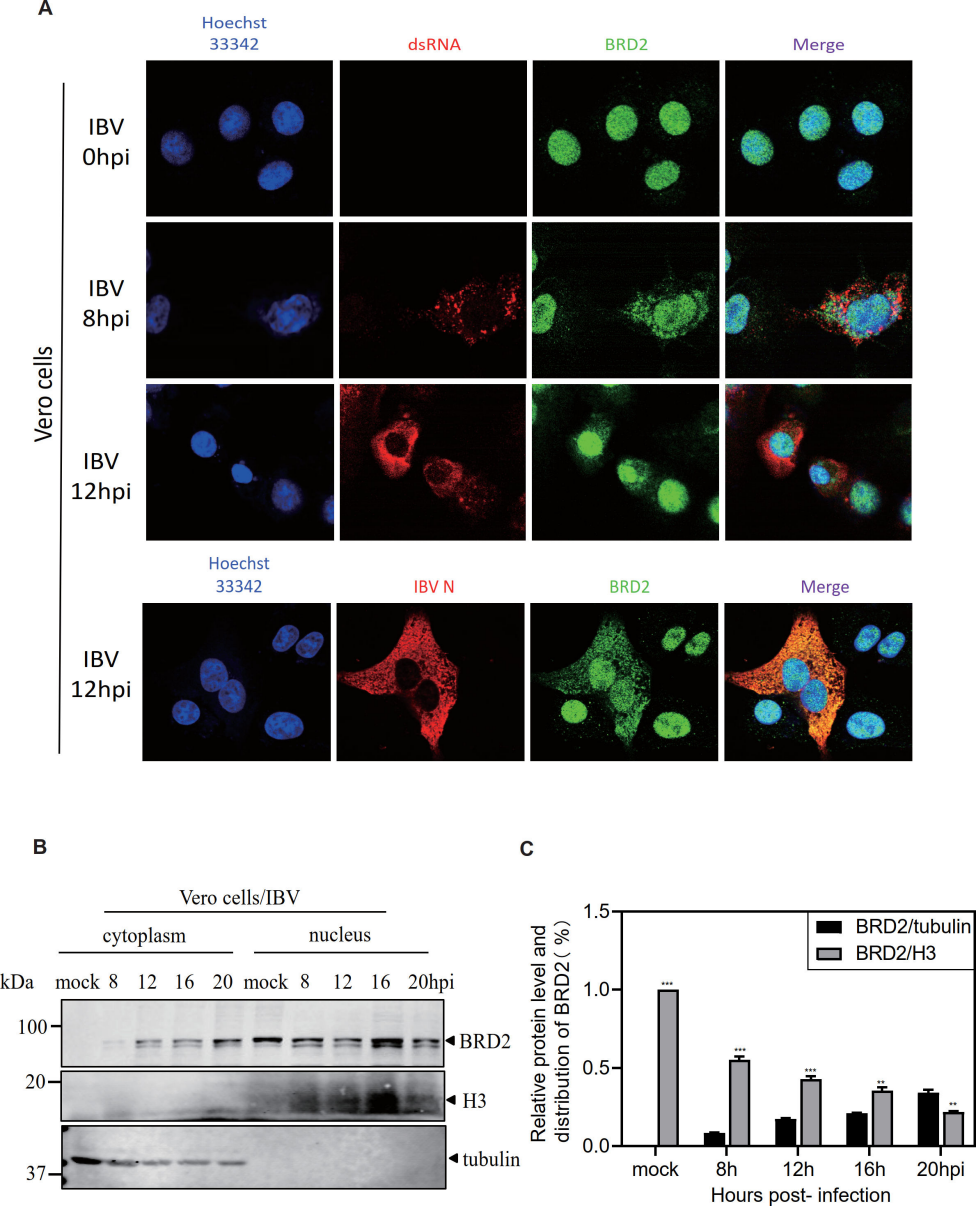
Re-localization of BRD2 from the nucleus to the cytoplasm in CoV-infected cells. (**A**) Cells infected with IBV (MOI~2) or mock-treated (UV-inactivated virus) were fixed at 0, 8, and 12 hpi, permeabilized, and immunostained with rabbit anti-BRD2 and mouse anti-IBV N/dsRNA. IBV N/dsRNA (Alexa Fluor 488, green), BRD2 (Alexa Fluor 594, red), and nuclei (DAPI/Hoechst 33,342, blue) were visualized. (**B**) Vero cells were either infected with IBV at an MOI~2 or mock-treated with UV-inactivated IBV and harvested at the indicated times post-infection. Nuclear and cytosolic fractions were prepared and subjected to western blotting with the indicated antibodies. Sizes of protein ladders in kDa were indicated on the left. (**C**) The grayscale values of protein bands shown in panel **B** were measured using Image J. The relative protein expression level of BRD2 in the nuclear fraction of the mock group was set as 100%, and the levels in other groups were calculated as percentages relative to this value.

Immunofluorescence and confocal experiments were then performed to determine the effect of IBV infection on the subcellular localization of BRD2. In uninfected Vero cells, BRD2 protein was predominantly localized in the nucleus (Fig. 3A). In Vero cells infected with IBV at 8- and 12 h post-infection, a minor but significant proportion of BRD2 proteins was translocated to the cytoplasm, although the majority of BRD2 remained in the nucleus (Fig. 3A). Staining of the infected with antibodies against viral double-stranded RNA (dsRNA) showed exclusively cytoplasmic localization, but without observable overlapping with BRD2 (Fig. 3A). Interestingly, partial co-localization of BRD2 with IBV N protein was observed in IBV-infected Vero cells at 12 h post-infection (Fig. 3A), suggesting that the cytoplasm-retained BRD2 may be recruited to the assembling virions (Fig. 3A), potentially via its association with E protein.

The subcellular localization and cytoplasmic retention of BRD2 in IBV-infected Vero cells were further studied by nuclear and cytoplasmic fractionation, and the relative levels of BRD2 protein presented in these fractions were quantified by densitometry analysis of the band densities on the Western blot gels with Image J software. The data showed that the relative protein level of BRD2 in the cytoplasm exhibited a gradual upward trend from 8.4% at 8 h post-infection to 34.2% at 20 h post-infection (Fig. 3B and C). On the contrary, the relative level of BRD2 protein in the nuclear fraction was reduced from 55.1% to 21.8% at the same time points (Fig. 3B and C). In consistency, the ratios of cytoplasmic/nuclear BRD2 protein were changed from below 15.3% at 8 h post-infection, increased to 40.3% and 58.9% at 12 and 16 h post-infection, and reached the peak ratio of 157.1% at 20 h post-infection. Taken together, these results demonstrate that a certain proportion of BRD2 protein is retained in the cytoplasm in IBV-infected cells, probably through its interaction with the E protein.

### Loose association of BRD2 with partially purified virions and interaction of IBV E protein with the endogenous BRD2

**Fig 4:**
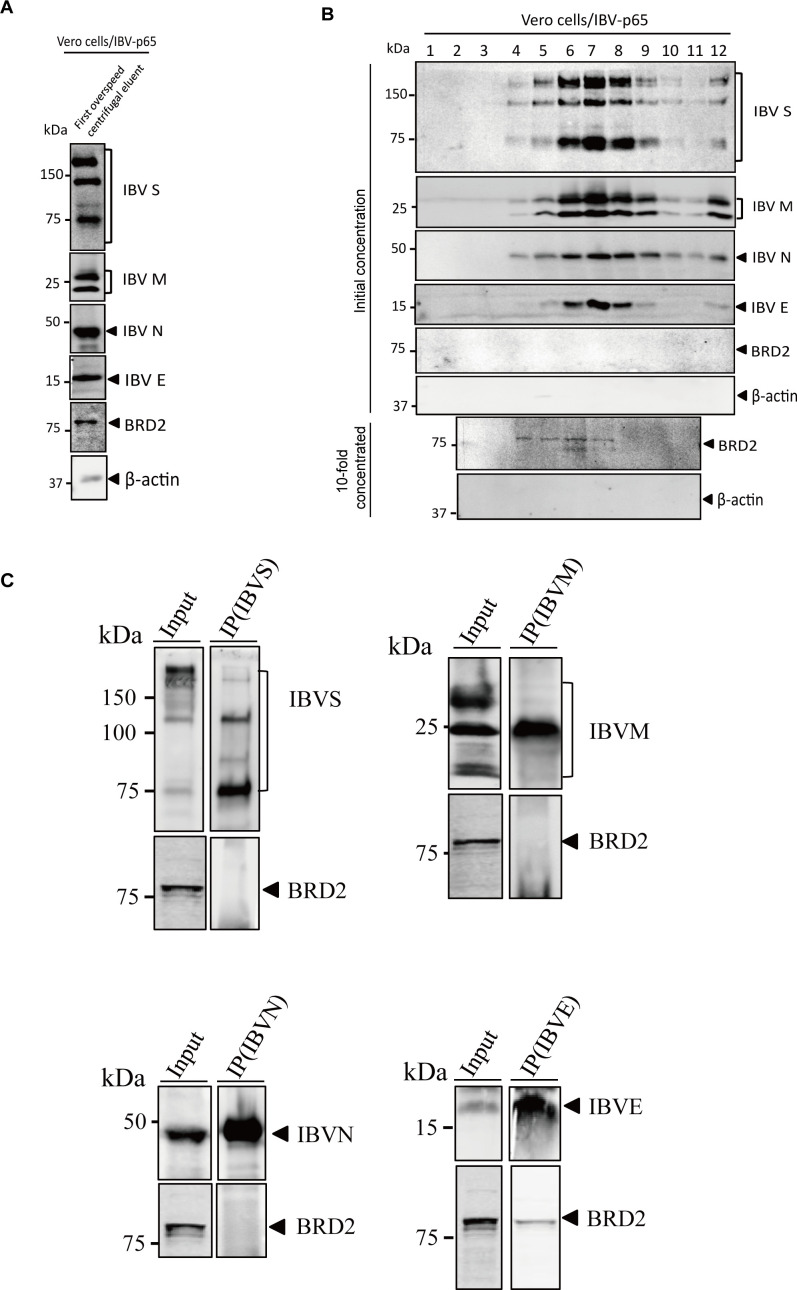
The association between the host protein BRD2 and IBV virions. (**A**) IBV-infected Vero cell supernatants were purified by sucrose gradient 20%. The sucrose pellet was analyzed by Western blot with the indicated antibodies. β-actin served as a loading control; protein ladder sizes (kDa) are shown. (**B**) IBV-infected Vero cell supernatants were purified by sucrose gradient (20%–60%). Fractions (12 total) were analyzed by Western blot with the indicated antibodies. Nine of twelve fractions (lanes 2–10) were 10× concentrated and analyzed by Western blot (antibodies indicated). β-actin was the loading control; protein ladder sizes in kDa are marked. (**C**) IBV-infected Vero cell supernatants were purified by sucrose gradient 20%. The sucrose pellet was immunoprecipitated with anti-IBV S/N/M/E and probed with anti-IBV S/N/M/E or anti-BRD2. Sizes of protein ladders in kDa were indicated on the left.

The possibility that the cytoplasm-retained BRD2 may be recruited into assembling virions was then tested. IBV particles were first precipitated and partially purified by spinning down through a 20% sucrose cushion and further purified by ultracentrifugation through a 20%–55% sucrose gradient. The results showed that the binding of BRD2 protein to the viral pellets was obviously detected after the first round of precipitation and partial purification through the sucrose cushion (Fig. 4A). After purification through the sucrose gradient, however, the presence of BRD2 protein in the fractions containing the majority of the viral particles was not significantly enriched (Fig. 4B). Fractions 2–10 were then 10-fold concentrated by ultracentrifugation, and the presence of BRD2 protein in lanes 4-7 was weakly detectable, with no significant difference in the amount of BRD2 protein among these lanes (Fig. 4B).

Co-IP experiments using the concentrated IBV virions precipitated and partially purified through the 20% sucrose cushion were performed. Results from this physiologically relevant setting demonstrate a specific interaction between the endogenous BRD2 and E protein, but not with the other three structural proteins, S, N, or M (Fig. 4C). As coronavirus E protein was not a known nuclear protein, these results suggest that the portion of BRD2 protein translocated to the cytoplasm in IBV-infected cells interacts with the E protein, attributable to its loose association with the viral particles.

### BRD2 protein regulates CoV replication and apoptosis

**Fig 5:**
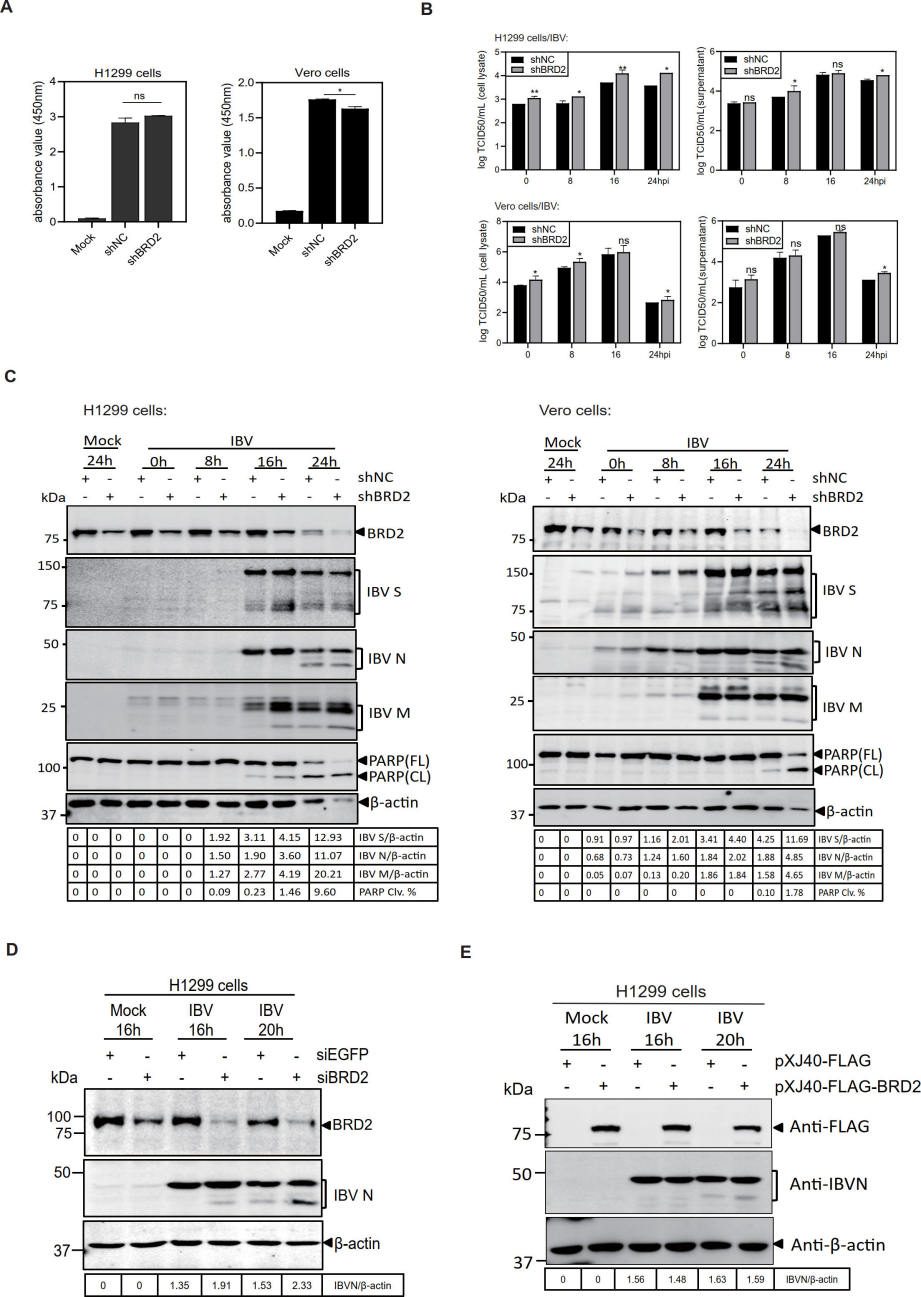
BRD2 protein regulates CoV replication and apoptosis. (**A**) Cell viability of H1299-shNC/BRD2 and Vero-shNC/BRD2 cells was assessed by CCK-8 assay (A450). Significance levels were presented by the *p*-value (ns, non-significant; *, *P*<0.05; **, *P* < 0.01; ***, *P* < 0.001). N.D., non-determined. (**B**) IBV titers (log TCID50/ml) in lysates/supernatants of infected H1299-shNC/BRD2 and Vero-shNC/BRD2 cells at the indicated time points. Significance as described for panel **A**. (**C**) Western blot analysis of IBV-infected (MOI ~2) or mock-treated H1299-shNC/BRD2 and Vero-shNC/BRD2 cells using the indicated antibodies. Beta-actin was included as the loading control. Sizes of protein ladders in kDa were indicated on the left. (**D**) IBV-infected or mock-infected H1299 cells pre-transfected with siEGFP/siBRD2 were analyzed by Western blot. Beta-actin was included as the loading control. Sizes of protein ladders in kDa were indicated on the left. (**E**) Western blot of IBV-infected or mock-infected H1299 cells transfected with pXJ40-FLAG/pXJ40-FLAG-BRD2. Beta-actin was included as the loading control. Sizes of protein ladders in kDa were indicated on the left.

To study the biological function of BRD2 in the regulation of CoV infection, two BRD2-knockdown stable clones, H1299-shBRD2 and Vero-shBRD2, together with their corresponding negative control cell clones H1299-shNC and Vero-shNC, were generated with short hairpin RNA targeting BRD2 (shBRD2) from H1299 and Vero cells, respectively. The proliferative activity of these four stable cell lines was assessed using the CCK8 cell proliferation activity detection kit. Compared with the negative control, the proliferative activity of H1299-shBRD2 stable cell line showed no significant difference, while the proliferative activity of Vero-shBRD2 stable cell line was marginally lower (Fig. 5A).

The effect of BRD2-knockdown on IBV replication was then assessed by infection with IBV at an MOI~ 2 and measuring TCID50 in cell lysates and supernatants, respectively, at 0, 8, 16, and 24 h post-infection. Compared with that in shNC stable cell lines, the viral titers in H1299-shBRD2 and Vero-shBRD2 stable cells were significantly increased in cell lysates and in the supernatants, though the difference was not as pronounced as in the cell lysates (Fig. 5B). These results demonstrate that knockdown of BRD2 promotes the replication of IBV in cells but renders less effect on the release of viral particles. The effect of BRD2-knockdown on IBV replication was further analyzed by time-course infection experiments. Compared with that in the control cell clones, the expression of structural proteins S, N, and M was higher in H1299-shBRD2 and Vero-shBRD2 stable cell lines infected with IBV (Fig. 5C). Moreover, IBV infection-induced apoptosis was significantly higher in the stable cell lines over the course of infection, compared with that in the control clones (Fig. 5C).

The effects of BRD2 knockdown on IBV replication and apoptosis were further analyzed by overexpression and transient knockdown of BRD2 using small siRNA, respectively. The protein expression level of IBV N proteins was increased in the transient BRD2-knockdown cells infected with IBV (Fig. 5D). However, overexpression of BRD2 did not significantly affect IBV replication, as suggested by the comparable levels of N protein relative to the vector control (Fig. 5E).

Similarly, in PEDV, HCoV-229E, and HCoV-OC43 infected-H1299-shBRD2 stable cell lines or Vero-shBRD2 stable cell lines, the expression of structural protein N was also significantly increased compared with that in control shNC stable cells (Fig. S2A and B). Significant induction of apoptosis was also observed during HCoV-229E and HCoV-OC43 infection of the knockdown cell clones, compared with that in the control clones (Fig. S2B). However, the level of apoptosis was not significantly affected in PEDV-infected H1299-shBRD2 and Vero-shBRD2 stable cell clones, compared with the shNC stable cell lines (Fig. S2A). In transient BRD2-knockdown cells infected with PEDV, HCoV-229E, and HCoV-OC43, the increased viral replication was also observed (Fig. S2C). Once again, overexpression of BRD2 also rendered an undetectable effect on the replication of PEDV, HCoV-229E, and HCoV-OC43 (Fig. S2D). These results confirm that BRD2 knockdown consistently enhanced the replication of four coronaviruses across three genera and induced increased late-stage apoptosis in multiple infected cells. Although the direct effect on replication is modest, BRD2 likely acts as a component in a broader host network supporting viral fitness, where individual contributions may be subtle but collectively important. This model aligns with the more pronounced role of the BRD2-E interaction in modulating immune signaling, which may constitute its primary function during infection.

### BRD2 promotes the induction of pro-inflammatory response in CoV-infected cells

**Fig 6:**
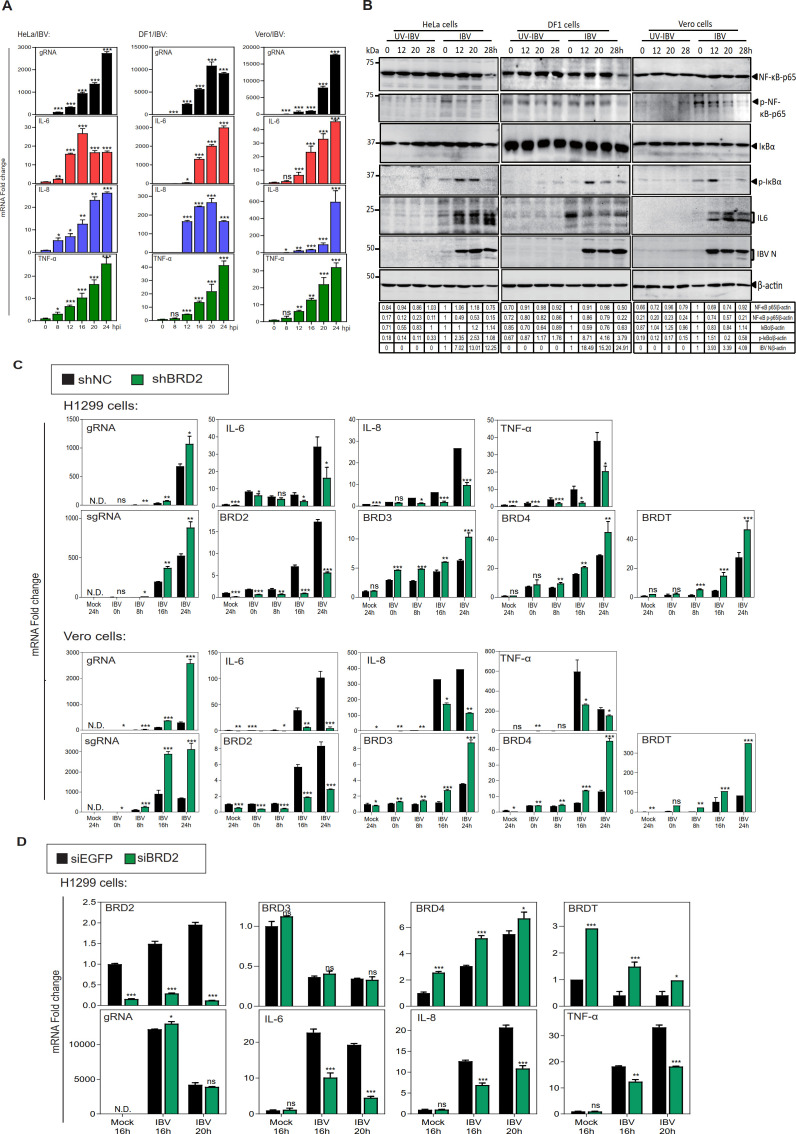
BRD2 promotes the induction of pro-inflammatory response in CoV-infected cells. (**A**) IBV-infected (MOI ~2) or mock-treated HeLa, DF1, and Vero cells were analyzed by RT-qPCR for viral gRNA and cytokine mRNAs (IL-6, IL-8, TNF-α), normalized to 0 hpi GAPDH. Significance levels were presented by the *P*-value (ns, non-significant; *, *P*<0.05; **, *P* < 0.01; ***, *P* < 0.001). N.D., non-determined. (**B**) Western blot analysis of IBV-infected or mock-treated HeLa, DF1, and Vero cells at the indicated time points. Beta-actin was included as the loading control. Sizes of protein ladders in kDa were indicated on the left. (**C**) RT-qPCR analysis of IBV RNAs (gRNA, sgRNA), cytokines (IL-6, IL-8, TNF-α), and BET family mRNAs (BRD2/3/4, BRDT) in H1299/Vero-shNC/BRD2 cells, normalized to GAPDH in shNC at 0 hpi. Significance as described for panel **A**. (**D**) IBV-infected or mock-infected H1299 cells pre-transfected with siEGFP/siBRD2 were analyzed by RT-qPCR for viral gRNA, cytokines, and BET mRNAs, normalized to GAPDH in siEGFP mock at 16 hpi. Significance as described for panel **A**.

BRD2 is an epigenetic factor containing a bromodomain that promotes mRNA expression by binding to promoters of related pro-inflammatory factor transcripts, and cytokines IL-6, IL-8, and TNF-α were selected for analysis based on their significant upregulation in our published transcriptomic data and their established roles as canonical BRD2 transcriptional targets (34). The functional involvement of BRD2 in CoV infection was then assessed by analyzing the mRNA and protein levels of these pro-inflammatory factors in IBV-infected HeLa, DF1, and Vero cells, three susceptible cell lines of human, avian, and monkey origins. The mRNA expression of IL-6, IL-8, and TNF-α was significantly induced by more than 20-fold at the late stages of IBV infection (Fig. 6A), and the phosphorylated forms of NF-κB p65 and IκBα kinases were obviously increased during IBV infection (Fig. 6B). The protein expression of IL-6 was also significantly induced by IBV infection (Fig. 6B). These data suggest that IBV infection activates the NF-κB signaling pathway and induces the expression of pro-inflammatory cytokines.

The impact of BRD2-knockdown on the induction of these pro-inflammatory factors was then studied by time-course infection experiments in IBV-infected BRD2-knockdown cell clones. Once again, the replication of IBV was significantly increased by BRD2-knockdown, as demonstrated by the significantly increased detection of IBV gRNA and sgRNA in BRD2-knockout cells (Fig. 6C). Significantly reduced mRNA levels of IL-6, IL-8, and TNF-α were detected in IBV-infected H1299-shBRD2 and Vero-shBRD2 cells (Fig. 6C). Notably, the transcription levels of other members of the BET family genes, including BRD3, BRD4, and BRDT, were significantly elevated in BRD2-knockdown cell lines, compared to shNC cell lines (Fig. 6C). Meanwhile, H1299 cells were transfected with siRNA duplexes targeting EGFP (negative control) and siBRD2, respectively, before being infected with IBV. Compared with siEGFP control, the mRNA levels of IL-6, IL-8, and TNF-α were significantly reduced in transient BRD2-knockdown cells infected with IBV (Fig. 6D). Transient knockdown of BRD2 also increased the transcription levels of BRD4 and BRDT, but BRD3 was less affected (Fig. 6D).

Similarly, analysis of the impact of BRD2-knockdown on the induction of pro-inflammatory factors during the time-course infection of PEDV, HCoV-229E, and HCoV-OC43 showed increased replication of PEDV, HCoV-229E, and HCoV-OC43 (Fig. S3A and B), and significantly reduced induction of IL-6, IL-8, and TNF-α in PEDV-infected H1299-shBRD2 and Vero-shBRD2 cells, as well as in H1299-shBRD2 cells infected with HCoV-229E and HCoV-OC43 (Fig. S3A and B). The transcription levels of other members of the BET family genes were also significantly elevated in these infected cells (Fig. S4). Similar results were also observed in transient BRD2-knockdown cells infected with PEDV, HCoV-229E, and HCoV-OC43, respectively, whereas some BET family genes showed a moderate increase (Fig. S4).

Overall, these results demonstrate the functional involvement of BRD2 in promoting viral replication and inflammatory responses in CoV-infected cells. As the transcription levels of other BET family gene members were significantly induced in BRD2-knockdown cells infected with these CoVs, this suggests the existence of a functional compensatory mechanism among BET family genes.

### BRD2 protein promotes the activation of NF-κB signaling pathway and the expression of pro-inflammatory factors

**Fig 7:**
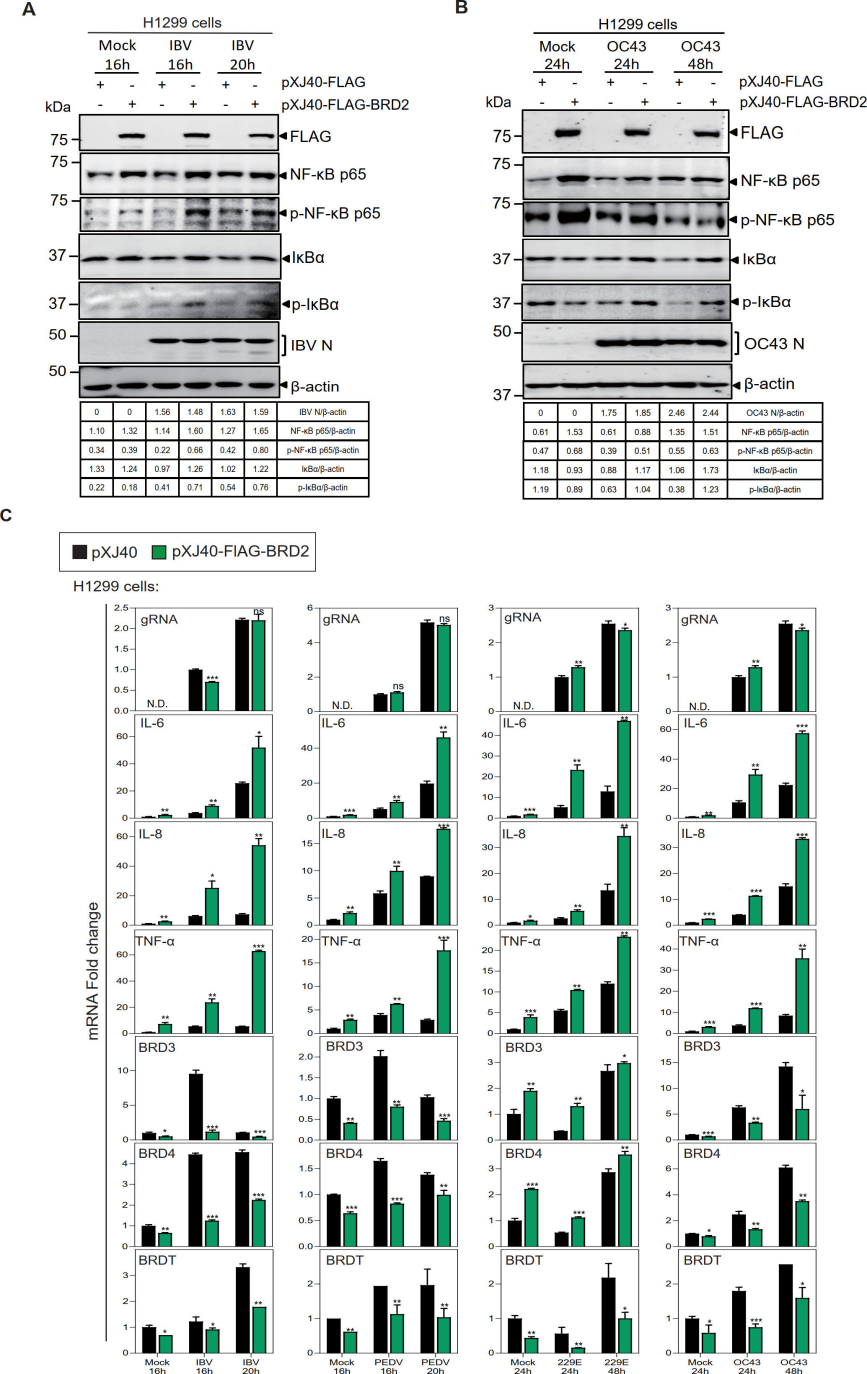
BRD2 protein promotes the activation of the NF-κB signaling pathway and the expression of pro-inflammatory factors. (**A**) H1299 cells transfected with pXJ40-FLAG or pXJ40-FLAG-BRD2 were infected with IBV or mock-treated, then analyzed by Western blot. Beta-actin was included as the loading control. Sizes of protein ladders in kDa were indicated on the left. The FLAG, IBV N, and β-actin loading control shown here are from the same experimental batch as the samples in Fig. 5E and are reused to ensure a consistent baseline for cross-figure comparison. (**B**) As in panel **A**, but infected with HCoV-OC43. Beta-actin was included as the loading control. Sizes of protein ladders in kDa were indicated on the left. (**C**) H1299 and Vero cells transfected as described for panel **A** were infected with IBV, PEDV, HCoV-OC43, or HCoV-229E. RT-qPCR measured viral gRNA, cytokine mRNAs (IL-6/8, TNF-α), and BET family transcripts (BRD2/3/4, BRDT), normalized to FLAG-transfected mock 16 hpi GAPDH. Significance levels were presented by the *P*-value (ns, non-significant; *, *P*<0.05; **, *P* < 0.01; ***, *P* < 0.001). N.D., non-determined.

To complement the loss-of-function experiment, BRD2 was cloned into a eukaryotic expression vector pXJ40-FLAG and transiently expressed in H1299 before being infected with IBV, PEDV, HCoV-229E, and HCoV-OC43, respectively. Overexpression of BRD2 in IBV and HCoV-OC43-infected H1299 cells increased the protein expression level of NF-κB p65, IκBα, and their respective phosphorylated forms (Fig. 7A and B). Significant increase detection of IL-6, IL-8, and TNF-α at the mRNA level was also found in H1299 cells infected with IBV, PEDV, HCoV-229E, and HCoV-OC43, respectively (Fig. 7C). Compared with in cells transfected with the empty vector control, the transcription levels of BRD3, BRD4, and BRDT genes were significantly lower than in the infected H1299 cells overexpressing BRD2 (Fig. 7C). However, in BRD2-overexpressing H1299 cells infected with HCoV-229E, BRDT was also significantly decreased at the mRNA level, while the transcription levels of BRD3 and BRD4 were significantly increased (Fig. 7C). These results suggest that BRD2 upregulates mRNA levels of IL-8 and other pro-inflammatory response-related genes, possibly by activating the NF-κB signaling pathway or by binding to promoters in the transcripts of these genes to promote mRNA expression.

The effects of BRD2 and E protein on the pro-inflammatory response were studied by co-expressing BRD2 and IBV E in 293T cells and treating with toll-like receptor (TLR3) agonist poly(I:C) and TLR4 agonist lipopolysaccharide (LPS), respectively. Total RNA was extracted at 24 h post-treatment, and the expression of related pro-inflammatory factors was analyzed by RT-qPCR. In cells treated with poly(I:C) and LPS groups, overexpression of BRD2 and IBV E on their own or together all significantly increased the expression levels of IL-6, IL-8, and TNF-α, compared with the negative control group transfected with the empty vector (Fig. S5). Notably, significantly higher expression of IL-6, IL-8, and TNF-α was detected in 293T cells co-expressing BRD2 and IBV E together than that in cells overexpressing BRD2 and IBV E protein alone (Fig. S5). These results demonstrate that the interaction of the BRD2 protein with the IBV E protein may synergistically promote the induction of related pro-inflammatory factors.

## DISCUSSION

**Fig 8:**
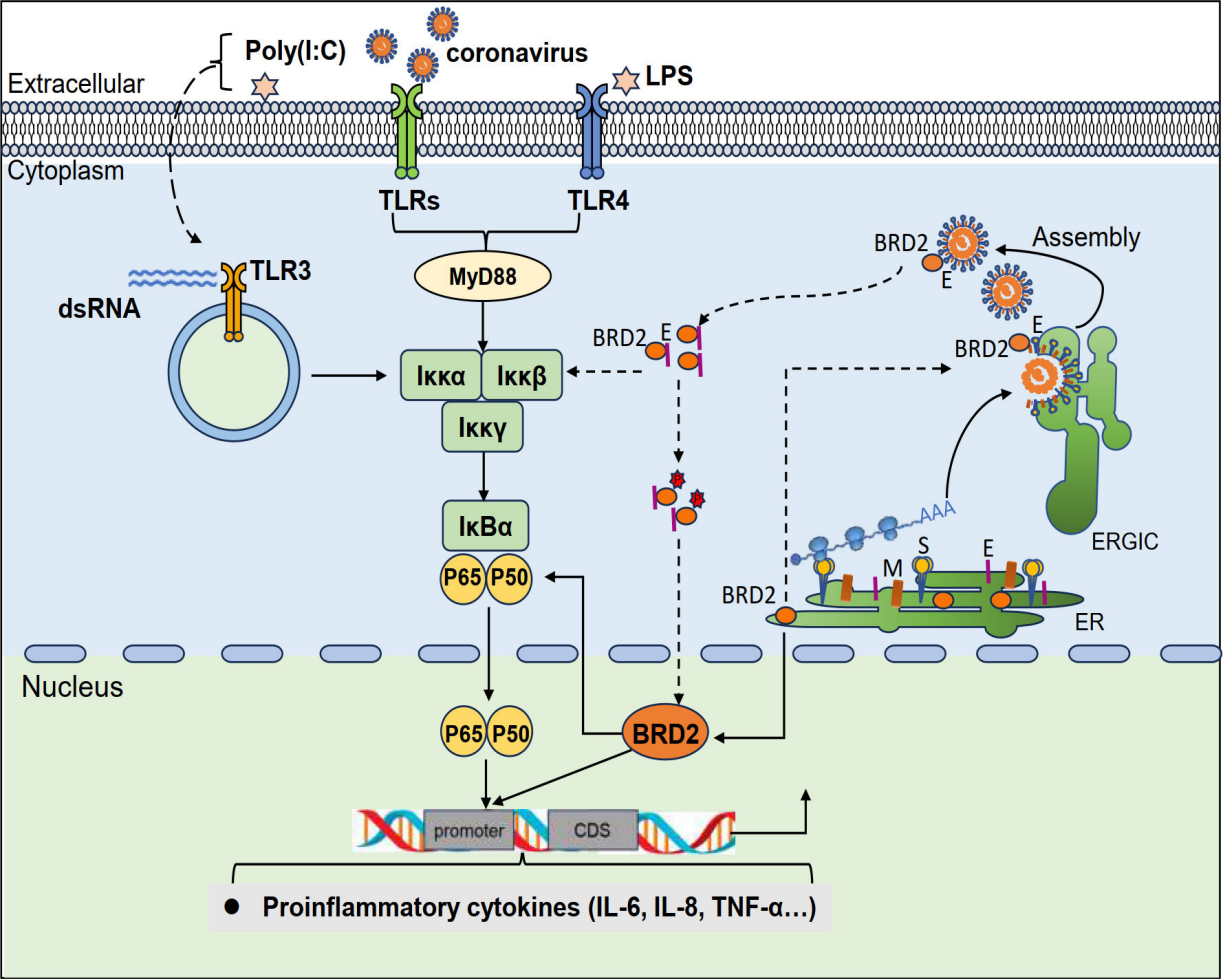
Diagram illustrating the current working model. Working model of BRD2-IκBα-NF-κB axis activation of pro-inflammatory cytokines/chemokines and BRD2-E protein interaction during CoV infection. Arrowheads indicate activation; blunt ends indicate suppression. Dashed lines represent incompletely characterized processes.

Severe CoV infection may trigger the release of elevated levels of pro-inflammatory cytokines and other inflammatory mediators, ultimately culminating in cytokine storms (35). This uncontrolled, hyper-inflammation would cause multiple organ damage, leading to organ failure and even death (36). It is evident that the inflammatory response triggered by these pro-inflammatory cytokines and other inflammatory mediators plays a crucial role in CoV infection and pathogenesis. In this study, we confirm the interaction of BET family protein BRD2 with the E protein from IBV, SARS-CoV, and SARS-CoV-2. By either directly binding to the promoters of these pro-inflammatory cytokine genes or activating the IκBα-NF-κB signaling pathway, BRD2 promotes the transcription of a number of important pro-inflammatory cytokines during CoV infection (Fig. 8). Interaction of BRD2 with CoV E plays a further, synergistic role in regulating the expression of these pro-inflammatory cytokines (Fig. 8).

Cytokine storm, the hallmark of severe COVID-19, arises from immune dysregulation and excessive cytokine production, driving hyperinflammation (37). SARS-CoV-2 infection is known to trigger a robust inflammatory response mediated by interleukins (such as IL-1, IL-6, IL-17, and IL-18), IFN-γ, TNF-α, TGF-β, and NF-κB (38). This is consistent with our observations that infection of cells with CoVs in three different genera significantly upregulated the expression of IL-6, IL-8, and TNF-α in cultured cells and induced the expression of NF-κB p65 and IκBα at the mRNA level as well as their phosphorylated forms at the protein level. The demonstration that a synergistic role was played by the interaction of BRD2 with the E protein from these CoVs in regulating the pro-inflammatory response further reveals a common pathophysiologic mechanism shared by many CoVs. Although the precise functional outcome requires further validation, this interaction correlates with the upregulation of inflammatory cytokines following infection and may potentially contribute to the dysregulation of the host immune response.

The mechanistic basis of BRD2 for this regulatory role involves both epigenetic mechanisms and activation of signaling pathways, either by typically binding to acetylated histones on cytokine promoters to regulate their transcription or by interacting with acetylated RelA in the NF-κB pathway to amplify NF-κB signaling and pro-inflammatory gene expression (39). In fact, BET family proteins are known to epigenetically modulate cytokine transcription, including pro-inflammatory and immune-related factors (40). Examples of this regulatory role in the scenario of virus infection include that the BET inhibitor apatarone suppresses SARS-CoV-2 RNA-induced expression of CXCL10, IL-6, and IL-1β in Calu-3 cells (41), downregulation of BRD3 in virus-infected macrophages impairs IFN-β production (42), and BRD4-NF-κB/RelA complexes mediate respiratory syncytial virus (RSV)-triggered inflammatory responses in airway epithelia (43). In Parkinson’s disease, α-synuclein induces BRD2 expression to drive neuroinflammation (44). In this study, we provide evidence that BRD2 overexpression enhanced the CoV infection-induced NF-κB activation and promoted IL-6 and IL-8 transcription, supporting the dual mechanisms of BRD2 in the regulation of pro-inflammatory response during CoV infection. However, the cytoplasmic sequestration of BRD2 during IBV infection and its role in enhancing the transcription of these cytokines appear to be contradictory. In fact, our study consistently showed that the total level of BRD2 protein was gradually reduced over the course of IBV and the other three coronavirus infections. In addition to the possibility that these virus infections might mediate its rapid degradation, the secretion of the portion of BRD2 protein loosely associated with the mature virions would be a main reason for the reduced detection of the total cellular BRD2 protein in the infected cells. Nevertheless, it is plausible that the cytoplasm-retained BRD2, via its interaction with E protein, may serve as a cofactor to induce robust host signaling, for example, NF-κB, as observed in this study, amplifying the cytokine response through a mechanism independent of its nuclear functions (45). Further functional studies are required to clarify this issue.

As a critical epigenetic regulator of mRNA transcription (46), growing evidence also implicates important regulatory roles of BRD2 and other BET family proteins in viral replication and pathogenesis (40). Knockdown of BRD2 was found to suppress human immunodeficiency virus (HIV) transcription as effectively as the BET inhibitor JQ1, whereas BRD4 knockdown exhibits weaker effects, highlighting the therapeutic potential of targeting BET proteins (47). Similarly, BET inhibitors can reduce African Swine Fever Virus (ASFV) replication in porcine macrophages (48). Intriguingly, BRD2 is essential for ACE2 transcription in human lung epithelial cells and cardiomyocytes, and BRD2 inhibitors effectively block ACE2 expression and SARS-CoV-2 infection in clinical models (24). BET proteins interact with viral proteins such as papillomavirus E2, herpesvirus LANA, gamma-retroviral integrase, and SARS-CoV-2 E (49). Our study expands this understanding by demonstrating that BRD2 specifically binds to the C-terminal domains of the E protein from IBV, SARS-CoV, and SARS-CoV-2. Notably, BRD2 knockdown enhanced the replication, viral release, and apoptosis induction of multiple CoVs (IBV, PEDV, HCoV-229E, HCoV-OC43), underscoring its pivotal role in modulating CoV infection.

The mechanism underlying the regulatory roles of BRD2 in CoV replication is yet to be revealed. Our findings demonstrate that BRD2 appears to play a more prominent role in the production of infectious IBV particles, compared to its limited influence on the release of these particles. The discrepancy between the infectious titer and physical particle release suggests that BRD2 may function primarily in a post-assembly step of the viral life cycle, potentially through mechanisms involving virion maturation that determine the viral infectivity. The lack of overlapping between BRD2 and viral double-strand RNA would exclude the possibility that the cytoplasm-retained BRD2 might physically bind to viral RNA and render a direct role in the regulation of the replication and transcription of viral genomic and subgenomic RNA. The loose association of BRD2 with IBV virions, however, would support the possibility that BRD2-E interaction may facilitate proper virion maturation and enhance the viral infectivity. As BRD2 is not directly associated with N and other structural proteins based on our Co-IP studies, the observed co-localization of IBV N with BRD2 in IBV-infected Vero cells would be the consequence of E-BRD2 interaction in the assembling virions.

While the data presented here demonstrate a compelling correlation between the BRD2-E interaction and the modulation of host immune responses, definitive establishment of a direct causal link requires further investigation. To unequivocally establish a direct link between the BRD2-E interaction and its functional outcomes, future work should focus on structure-guided mutagenesis. Identifying and characterizing specific mutants that disrupt this physical interaction while preserving the overall stability and function of both proteins would be critical. Comparing the effects of such binding-deficient mutants with their wild-type counterparts on the cytokine induction and viral replication may provide a more definitive mechanistic insight.

In conclusion, our findings establish BRD2 as a central modulator of host responses to CoV infection, regulating pro-inflammatory cytokines and innate immune genes through epigenetic and signaling pathways. The interaction between BRD2 and CoV E proteins, coupled with its impact on viral replication and cytokine storms, positions BRD2 as a promising therapeutic target. Future studies should explore how targeting BRD2 or its interplay with CoV E proteins could mitigate infection and hyperinflammation, offering new avenues for therapeutic intervention.

## Data Availability

All data generated or analyzed during this study are included in this published article and its supplemental material.

## SUPPLEMENTAL MATERIAL

Fig. S1 jvi.02201-25-s0001.tif Interaction of the C-terminal region of BRD2 protein with the C-terminal region of coronavirus E protein.

Fig. S2 jvi.02201-25-s0002.tif BRD2 protein regulates coronavirus replication and apoptosis.

Fig. S3 jvi.02201-25-s0003.tif BRD2 promotes the induction of pro-inflammatory response in coronavirus-infected cells.

Fig. S4 jvi.02201-25-s0004.tif BRD2 promotes the induction of pro-inflammatory response in coronavirus-infected cells.

Fig. S5 jvi.02201-25-s0005.tif Overexpression of BRD2 and IBV E proteins synergistically promotes pro-inflammatory response.

ASM does not own the copyrights to Supplemental Material that may be linked to, or accessed through, an article. The authors have granted ASM a non-exclusive, world-wide license to publish the Supplemental Material files. Please contact the corresponding author directly for reuse.
