# Supplementary figures and images for: Interaction of coronavirus E protein with BRD2 plays important regulatory roles in viral replication and induction of pro-inflammatory response
Source: J Virol. 2026 Mar 3;100(3):e02201-25. doi: 10.1128/jvi.02201-25 (PMC13011442; doi:10.1128/jvi.02201-25)

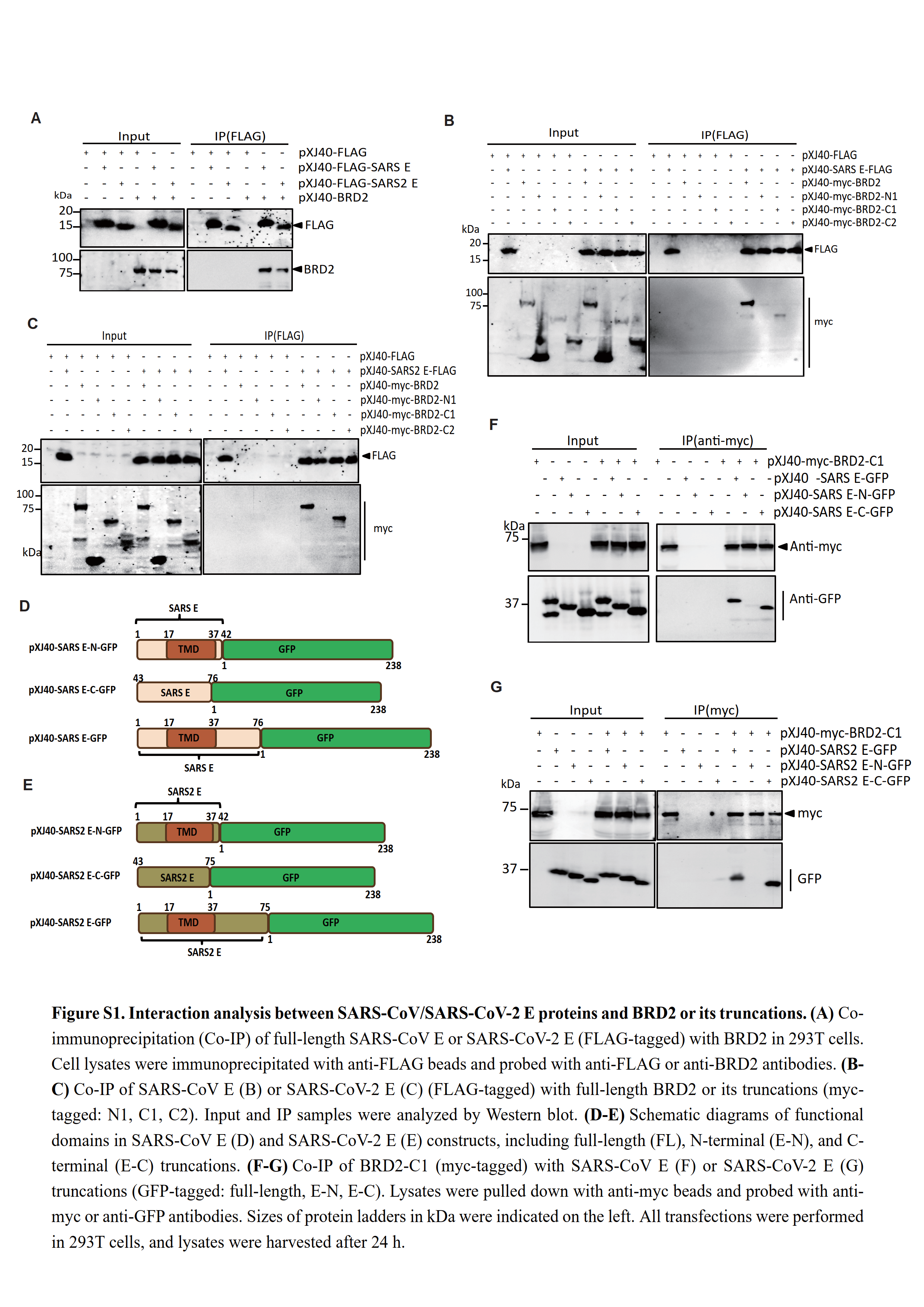

Supplement: Fig. S1 — Interaction of the C-terminal region of BRD2 protein with the C-terminal region of coronavirus E protein. [file jvi.02201-25-s0001.tif]

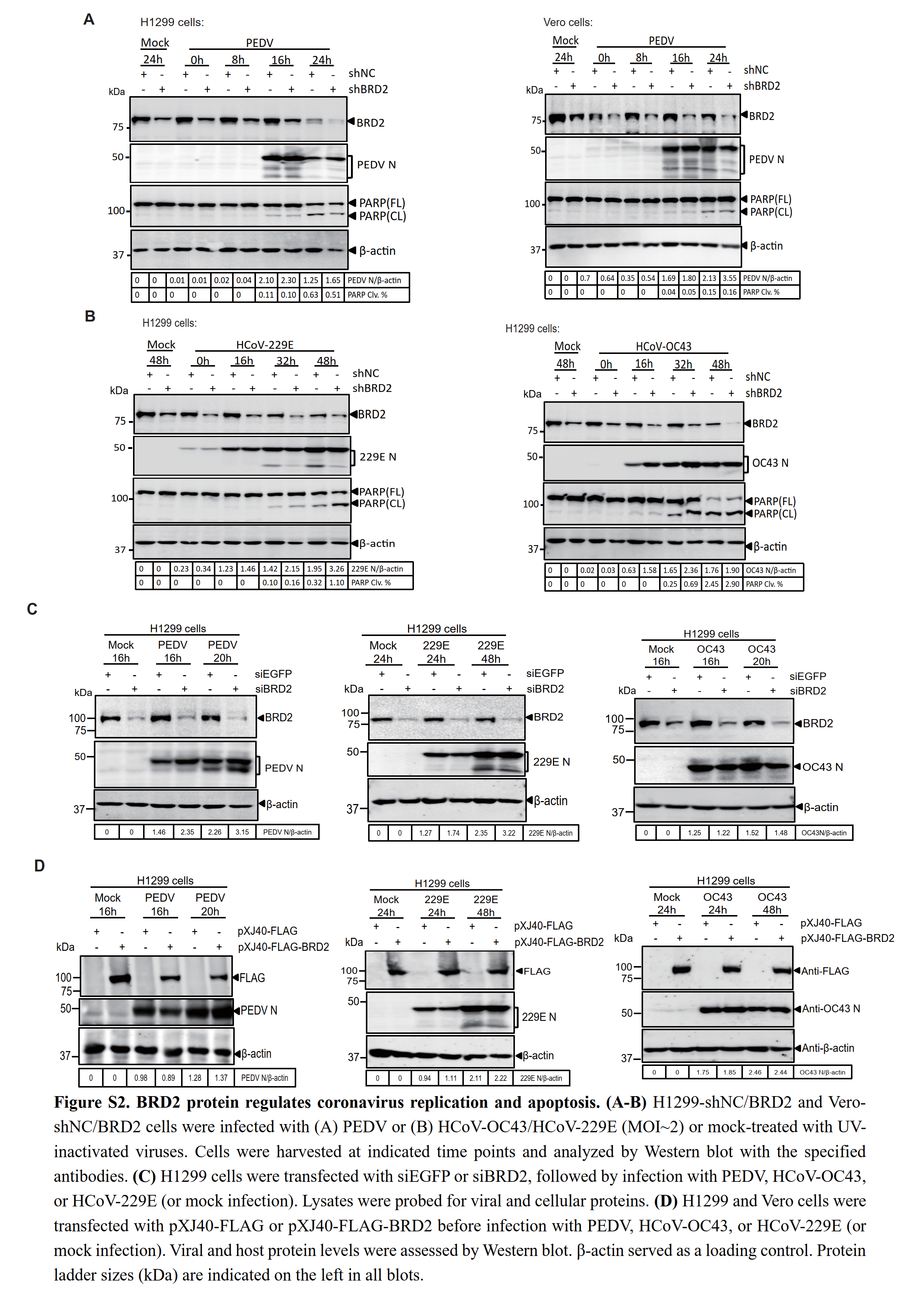

Supplement: Fig. S2 — BRD2 protein regulates coronavirus replication and apoptosis. [file jvi.02201-25-s0002.tif]

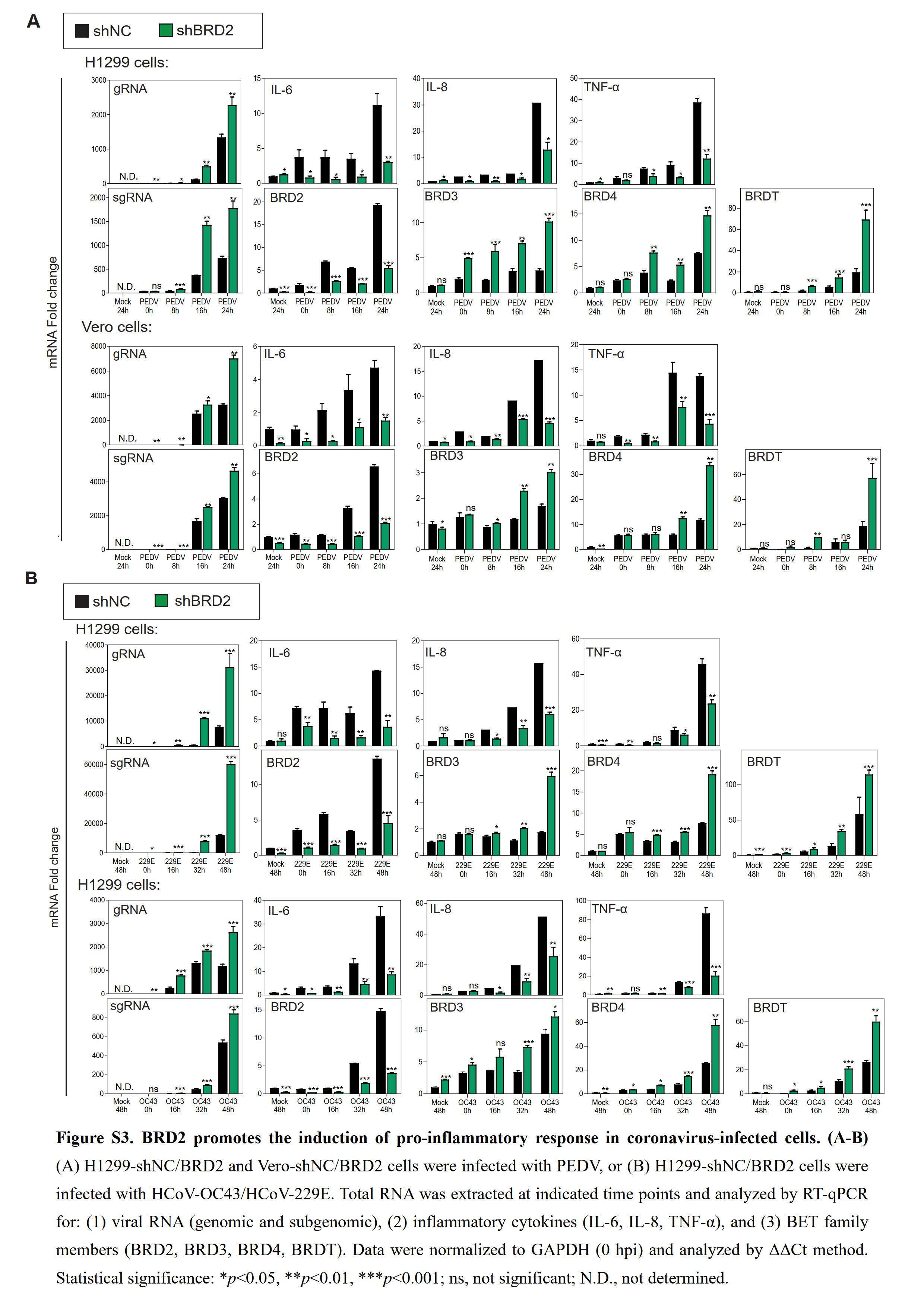

Supplement: Fig. S3 — BRD2 promotes the induction of pro-inflammatory response in coronavirus-infected cells. [file jvi.02201-25-s0003.tif]

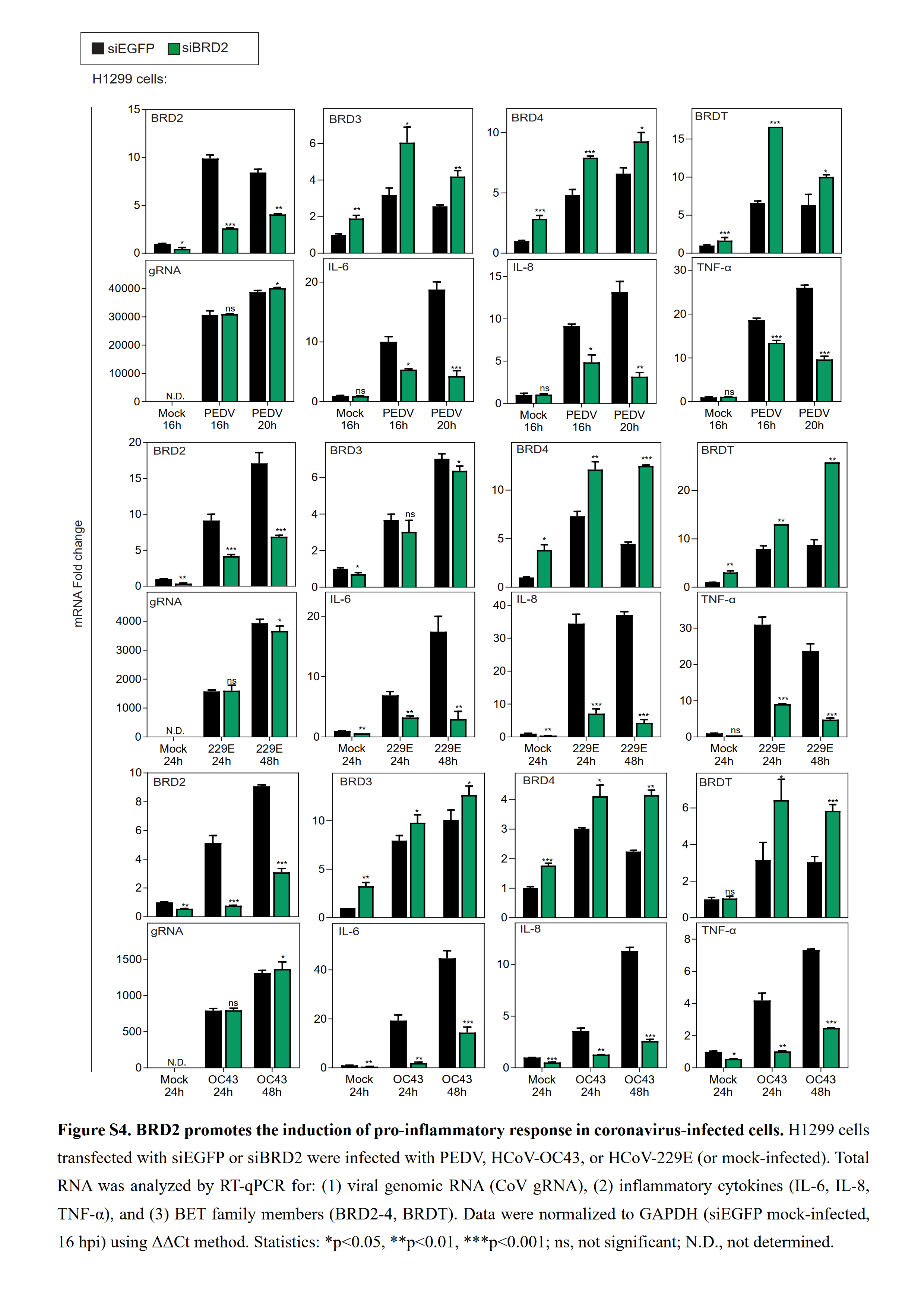

Supplement: Fig. S4 — BRD2 promotes the induction of pro-inflammatory response in coronavirus-infected cells. [file jvi.02201-25-s0004.tif]

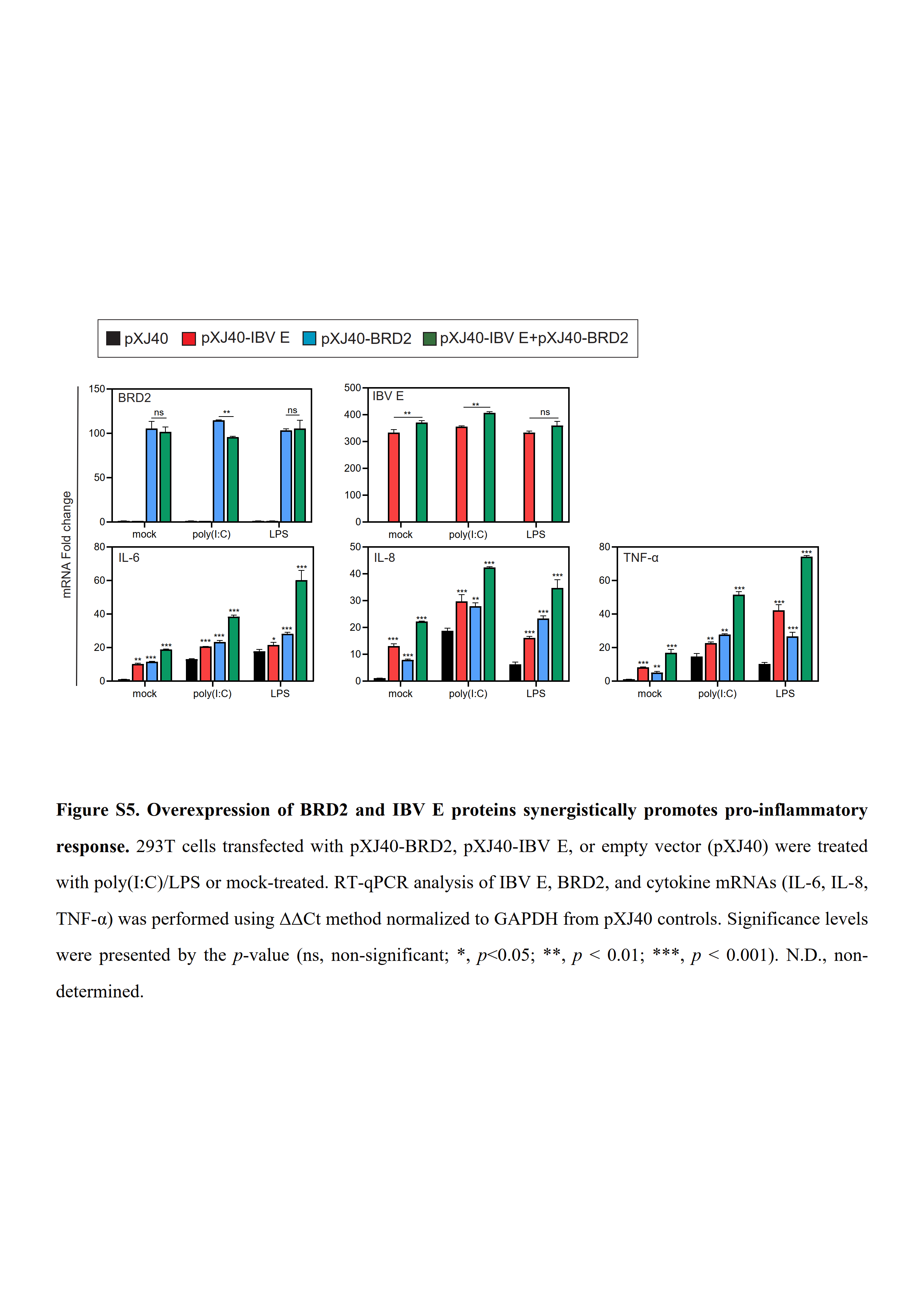

Supplement: Fig. S5 — Overexpression of BRD2 and IBV E proteins synergistically promotes pro-inflammatory response. [file jvi.02201-25-s0005.tif]
